# Autoamputation of the appendix and survival of the amputated part: a rare case report and literature review

**DOI:** 10.1186/s12893-022-01700-1

**Published:** 2022-06-27

**Authors:** Mingxiang Wang, Shili Ning, Yaqing Liu, Zhao Chen, Haodong Jiang, Shabnam Faiz, Fuwen Luo

**Affiliations:** 1 The Second Hospital of Dalian Medical University, Zhongshan Road, Shahekou District, Dalian, Liaoning People’s Republic of China; 2 Department of General Surgery, The Second Hospital of Dalian Medical University, Zhongshan Road, Shahekou District, Dalian, 116023 Liaoning People’s Republic of China

**Keywords:** Appendix, Appendicitis, Autoamputation, Laparoscopic appendectomy, Pseudo-duplication appendix

## Abstract

**Background:**

Autoamputation of the appendix, i.e., complete separation of a part of the appendix without any surgical intervention, has been rarely documented in the literature in recent years. Herein, we report a case where the amputated part of the appendix was viable after autoamputation and reviewed the related literature.

**Case presentation:**

A 39-year-old female patient was admitted to our hospital complaining of abdominal pain and subsequently underwent an emergency laparoscopic appendectomy (LA). Intraoperatively, we found an abnormally short appendix protruding from the cecum and a strip-like tissue attached to the mesoappendix, considered a duplex appendix, was resected. Finally, in conjunction with the histopathology findings and the past medical history, the patient was diagnosed with “Pseudo-duplication of the Appendix”.

**Conclusions:**

Autoamputation of the appendix resulting in preserved tissue viability and absence of necrosis at both ends, can be termed as “Pseudo-duplication of the Appendix”. This condition is very rare in clinical practice and has not been reported in China, to the best of our knowledge. It has been established that the autoamputated appendix can produce chronic inflammation, intestinal fistulae and even cancer, affecting the patient’s quality of life. Accordingly, a clear diagnosis and timely management are essential. In this report, we established a novel classification for “Pseudo-duplication of the Appendix”, hoping that our report will help surgeons better understand this anatomical anomaly of the appendix, to help during the differential diagnosis process and avoid confusion.

## Background

Autoamputation of the appendix which refers to the complete separation of any part (the tip, the body or the root) of the appendix without any surgical intervention [1], is rarely observed during clinical practice and is thought to result from full-thickness necrosis of a portion of the appendix due to tissue ischemia, which can cause severe diffuse peritonitis and even intestinal fistulas. Interestingly, in our case, the distal amputated end of the appendix was partially tethered to the mesoappendix with a preserved blood supply. Viable appendiceal tissue and a confined inflammatory response were observed, and both the proximal and distal ends of the autoamputated appendix spontaneously healed, which led to the diagnosis of “Pseudo-duplication of the Appendix”. In this paper, we systematically classified “Pseudo-duplication of the Appendix” and reviewed the published literature. To the best of our knowledge, this is the first case to be documented in China.

## Case presentation

A 39-year-old female patient presented to the outpatient clinic of the Second Hospital of Dalian Medical University with intermittent right lower abdominal pain and nausea for 7 days. Upon admission, physical examination revealed right lower abdominal tenderness, with no rebound tenderness or abdominal guarding, and the vital signs were: T: 36.7 °C, P: 72 beats/min, BP: 127/80 mmHg. The complete blood count showed mild leucocytosis (WBC count: 10.32 × 10^9^/L, normal range: 4–10 × 10^9^/L), suggestive of an infection. The patient previously underwent computed tomography (CT) scan of the abdomen in another hospital that showed inflammatory exudates in the ileocecal region and edema of the intestinal tract, while the appendix could not be visualized. The patient was subsequently transferred to our hospital for further consultation and treatment. The patient conceded that she experienced severe lower abdominal pain 2 years before but did not seek medical attention at that time and self-medicated with anti-inflammatory drugs. Her abdominal pain improved in about 5 days. Since then, she occasionally experienced dull pain in her right lower abdomen and self-medicated with oral antibiotics without seeking medical advice. Her past family and psycho-social history were unremarkable. After excluding surgical contraindications, the patient underwent emergency laparoscopic exploration.

After induction of general anesthesia, a pneumoperitoneum was established by the conventional three-port method, and the abdominal cavity was explored laparoscopically. We observed some adhesions between the small intestine and the lateral abdominal wall. After separating the adhesions, a small amount of pelvic fluid was seen, and no obvious abdominal and pelvic organ abnormalities were found. An appendix of about 2 cm in length and 1 cm in diameter protruded from the cecum during the operation with obvious edema, accounting for the patient’s right lower abdominal pain. After aspiration of the pelvic fluid, the appendix was removed.

**Fig. 1:**
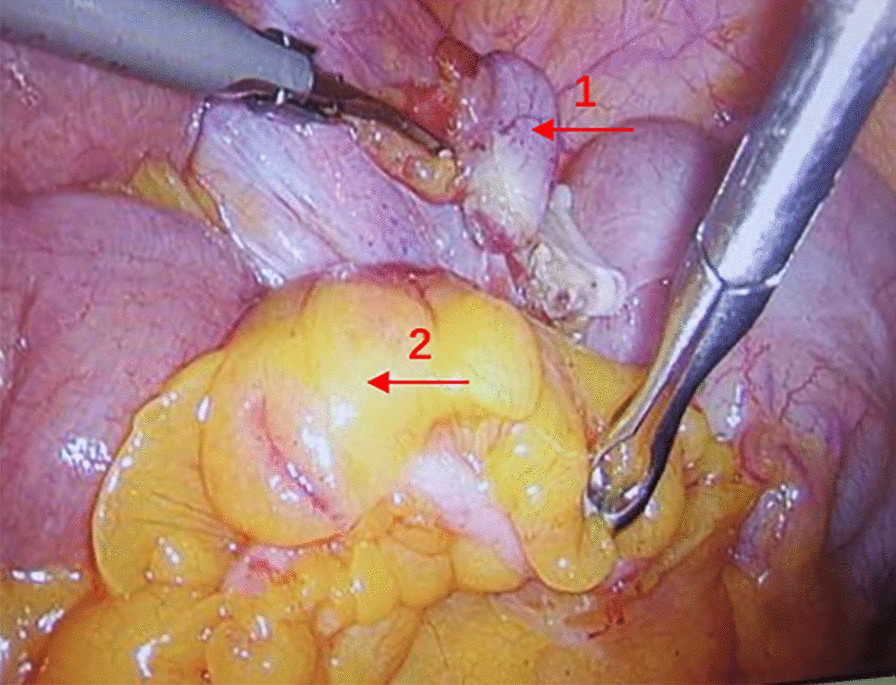
Laparoscopic exploration revealed a short appendix attached to the root of the cecum, about 2 cm in length and 1 cm in diameter, with obvious edema (arrow 1); and a strip-like tissue attached to the distal mesoappendix, approximately 4 cm in length and 0.5 cm in diameter (arrow 2)

Interestingly, further examination revealed a strip-like tissue attached to the distal mesoappendix, approximately 4 cm in length and 0.5 cm in diameter (Fig. 1). The intestinal tract and pelvic organs were carefully examined to exclude intestinal diverticulum, abdominal foreign objects and gynecological diseases. We suspected that the strip-like tissue suspected was a duplex appendix and dissected it along the tip to the end of the strip. Intriguingly, we found it was not connected to the surrounding intestinal tract and then resected it. After surgery, the surgical specimens were sent for histopathological examination.

**Fig. 2:**
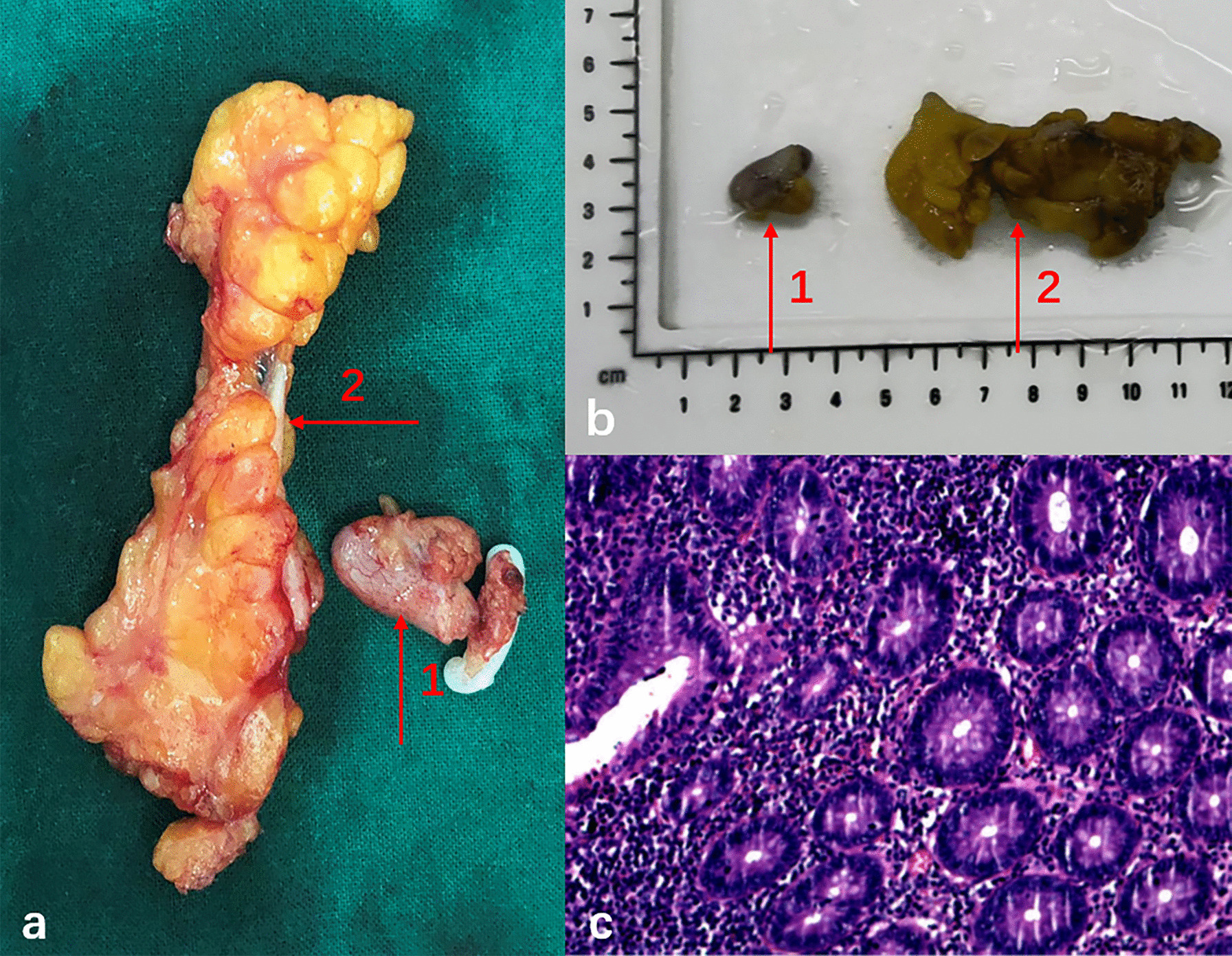
Surgical specimens: appendix 1 (short) was 1.6 cm in length, 0.8 cm in diameter with a smooth plasma membrane and visible blind end (arrow 1); appendix 2 (long) was 3 cm long, 0.4 cm in diameter with a smooth plasma membrane (arrow 2) (**a**,** b**). Pathological diagnosis: acute episode of chronic appendicitis with autoamputation of the appendix (**c**)

The postoperative histopathology showed that the excised short appendix and the strip-like tissue were appendiceal tissues. As seen in Fig. 2a, b, appendix 1 (short) was 1.6 cm in length, 0.8 cm in diameter with a smooth plasma membrane and visible blind end; appendix 2 (long) was 3 cm long, 0.4 cm in diameter with a smooth plasma membrane. The case was diagnosed as an acute episode of chronic appendicitis with autoamputation of the appendix (Fig. 2c).

After the operation, the patient received routine anti-inflammatory and rehydration treatment. Two days later, the patient passed gas with significant alleviation of the abdominal pain and was subsequently discharged. At 1-month follow-up after hospital discharge, the patient fully recovered without any gastrointestinal discomfort.

## Discussion

Acute appendicitis is one of the most common acute abdominal conditions, with an incidence of approximately 5.7–57/100,000 [2] and a high prevalence in children and adolescents. An increasing body of evidence suggests that the incidence of acute appendicitis is related to race, sex, age, obesity, season and altitude [3–5]. Importantly, appendicitis exhibits an irreversible and progressive phenotype leading to gangrene, perforation, and abscess formation. Accordingly, appendectomy is the standard treatment for appendicitis [2].

Appendicitis can be divided into acute and chronic appendicitis based on the clinical presentation, with acute appendicitis being more common in clinical practice. Acute appendicitis can be divided into four types depending on the severity: acute simple appendicitis, acute suppurative appendicitis, gangrenous/perforated appendicitis, and mucocele of the appendix. Of these, gangrenous/perforated appendicitis is the most severe form of appendicitis. It has been established that the appendiceal artery is a small terminal artery, which often leads to impaired blood flow due to inflammation and suppuration of the appendix, causing ischemic necrosis of the appendiceal tissue. This process leads to gangrene, perforation, appendiceal rupture and autoamputation in severe cases, causing diffuse peritonitis or mucocele of the appendix.

Initially, our surgical team assumed that our patient had a duplex appendix when we found that the distal atrophied part of the appendix was attached to the mesoappendix. However, we were convinced that this is a rare case of an anatomical anomaly of the appendix since our intraoperative findings (the distal part of the appendix attached to the mesoappendix was not connected to the surrounding intestinal tract) did not correspond to any type of duplex appendix in the Cave–Wallbridge classification [6]. Given that the patient experienced severe right lower abdominal pain 2 years ago and self-medicated, we hypothesized that the patient suffered from acute gangrenous appendicitis at that time. Appendiceal tissue ischemia led to full-thickness necrosis of the central part of the appendix, which eventually led to its complete severance. The inflammatory reaction was confined around the distal and proximal ends of the autoamputated appendix. As the inflammatory response evolved, the lesions at the severed ends of the appendix organized followed by the formation of scar tissue, which did not lead to diffuse peritonitis, and the patient’s condition gradually stabilized. In addition, the distal part of the appendix was still partially attached to the mesoappendix after appendiceal autoamputation, which maintained its blood supply and allowed the distal part of the appendix to survive. The distal part of the appendix was no longer attached to the intestinal tract and gradually atrophied, forming a tiny appendix of only 4 mm in diameter. The final diagnosis was “Pseudo-duplication of the Appendix”.

The online databases PubMed and ISI Web of Science were retrieved using medical subject headings (MeSH) terms and free-text words. The following search terms were applied: “autoamputation, auto-amputation, spontaneous amputation” and “appendix, appendicitis, appendectomy”. The full texts of each study were reviewed in detail, and the references of these texts were searched to identify further relevant studies. A total of 10 relevant papers were screened. Importantly, Judd et al. first documented “autoamputation of the appendix” in 1915 [1], followed by Wejsflog et al. [7], Iasocki et al. [8] and Volnohradský et al. [9], but no full text of the above articles were available. Sachs et al. reported a case of mucocele occupying the entire appendix, with laparoscopic findings of appendix autoamputated from the root [10]; Iuchtman et al. reported a total of 5 cases of appendiceal autoamputation over 30 years (1961–1991), no detailed description was provided [11]. Halvey et al. reported a 20-day-old male infant with a strangulated and gangrenous appendix within the right inguinal hernial sac, with a severe inflammatory reaction that led to autoamputation of two-thirds of the appendix [12]. Moreover, Lovrenski et al. reported a case of a 2-year-old girl with a free appendicolith detected by ultrasound suggestive of perforated appendicitis and an autoamputated appendix with its tip missing was found during laparoscopy [13]. Weil et al. reported a 2-year-old boy with a history of necrotizing enterocolitis presenting a calcified mass within the transverse mesocolon with no connection to the intestinal tract, which was pathologically confirmed to be appendiceal tissue. A final diagnosis of autoamputation of the appendix was established [14]. Markey et al. reported a 26-year-old female with autoamputation of the appendix secondary to chronic pelvic abscess involving an endometrioma and the right adnexa [15]. Importantly, the above studies suggest that appendiceal autoamputation can involve any part of the appendix, and the amputated part can be present in various locations, which is difficult to diagnose by non-invasive examinations. The presence of appendiceal autoamputation is confirmed intraoperatively or postoperatively in almost all cases. Autoamputation of the appendix has rarely been documented in recent years, and this is the first case of autoamputation of the appendix, with preserved tissue viability and absence of necrosis at both ends, reported in the literature.

In contrast to the classification of duplex appendix proposed by Cave–Wallbridge, the presence of a strip near the ileocecal region, similar in appearance to a duplicated appendix, can be termed “Pseudo-duplication of the Appendix”. This concept proposed by Goldschmidt et al. [16] was described by Khanna et al. as previous inflammation of the appendix that led to autoamputation leading to attachments to a new location such as the cecum, with a similar morphology to an appendix duplex [17, 18]. A literature review did not reveal a detailed generalization or classification of the pseudo-duplication appendix. We suggest that “Pseudo-duplication of the Appendix” should be classified into the following three types: Type A: appendiceal autoamputation due to various causes such as severe gangrenous perforated appendicitis, iatrogenic or non-iatrogenic injury, where the autoamputated end of the appendix is detached and attached to the ileocecal region or colon, and the strip formed after the inflammation subsides, i.e., the situation described in this case; Type B: intestinal diverticula near the ileocecal region, such as ileal diverticula, colonic diverticula, Meckel’s diverticula, etc. Some of the diverticula can be striated in appearance under the wrapping of the mesentery, and the symptoms and signs of diverticulitis near the ileocecal region are similar to those of appendicitis, which can easily lead to misdiagnosis; Type C: strip formed under the wrapping of the mesentery by abdominal foreign objects such as birth control rings. Preoperative tests should be completed and a careful history taken to clarify the diagnosis.

“Pseudo-duplication of the Appendix” is rarely reported clinically which may be attributed to poor awareness of clinicians as it is not a duplex appendix subtype. Such cases are difficult to detect on imaging due to atrophy of the autoamputated part of the appendix, which may only present with edema of the surrounding intestinal wall or even no positive findings, making it very easy to miss and misdiagnose. For such patients, a careful preoperative history is essential. Given that appendiceal autoamputation can involve any part of the appendix and the amputated part can be present in various locations, we recommend that the adjacent intestinal tract should be carefully explored before appendectomy, regardless of the surgical approach. If strips or omental wrapped adhesions are found near the adjacent intestinal tract, the adhesions should be carefully separated and completely skeletonized to avoid omissions. Besides, if an abnormally short appendix is observed intraoperatively, “Pseudo-duplication of the Appendix” should be highly suspected. The mucosa of the autoamputated appendix section still retains secretory functions and can cause chronic inflammation and affect the patient’s quality of life. Long-term inflammatory stimulation may even lead to the carcinoma of the appendix, emphasizing the need for timely diagnosis and treatment.

## Conclusions

Notwithstanding that autoamputation of the appendix is not an uncommon finding in clinical practice, both ends of the appendix rarely remain viable without necrosis; this condition can be termed as “Pseudo-duplication of the Appendix”. To the best of our knowledge, no cases have been reported in China, and we established a novel classification system. It is common practice that when appendiceal autoamputation occurs, the diseased appendix is almost always surgically removed at the first presentation. In some cases, few patients with appendiceal autoamputation do not undergo surgery and receive conservative treatment that resolves the symptoms. In such cases, the blood supply of the amputated end is preserved and remains viable, leading to “Pseudo-duplication of the Appendix”. Importantly, emphasis should be placed on detailed patient history. In patients with a history of severe right lower abdominal pain that was not treated surgically, presenting with recurrent lower abdominal pain and unclear imaging findings, the possibility of “Pseudo-duplication Appendix” should be considered. If an abnormally short appendix is found intraoperatively, the abdominal cavity should be carefully explored, especially the ileocecal region. Moreover, suppose intestinal adhesions or encapsulated strips are found. In that case, they should be carefully separated to identify to ensure that a pseudo-duplication appendix is not missed, which may result in serious clinical consequences, such as chronic inflammation, intestinal fistula, abdominal infection or even cancer. Surgeons should therefore be aware of anatomical anomalies and variants of the appendix for an accurate clinical diagnosis. Patients with a history of severe right lower abdominal pain not treated surgically, now complaining of recurrent lower abdominal pain, should be educated to seek medical attention to avoid serious consequences.

## Data Availability

All data generated or analyzed during this study are included in this published article.

## Abbreviations

LA: Laparoscopic appendectomy
T: Temperature
P: Pulse
BP: Blood pressure
WBC: White blood cell
CT: Computed tomography
